# The influence of rhizosphere soil microorganisms and environmental factors on gentiopicroside content in the roots and rhizomes of *Gentiana scabra* Bunge from Liaoning Province

**DOI:** 10.3389/fmicb.2025.1554981

**Published:** 2025-03-13

**Authors:** Jianming Hou, Haibo Yin, Dan Wang, Jiayi Luo, Wenqi Yang, Tingguo Kang

**Affiliations:** ^1^College of Pharmacy, Liaoning University of Traditional Chinese Medicine, Dalian, Liaoning, China; ^2^State Key Laboratory for Quality Ensurance and Sustainable Use of Dao - di Herbs, Beijing, China

**Keywords:** *Gentiana scabra* Bunge, rhizosphere soil microorganisms, gentiopicroside, environmental factors, association analysis

## Abstract

**Background:**

Rhizosphere soil microorganisms, as the second genome of plants, play an important role in the formation of secondary metabolites of medicinal plants and are one of the key factors in the formation of the authenticity of medicinal materials.

**Methods:**

In this paper, the rhizosphere soils of *Gentiana scabra* Bunge from six producing areas in Liaoning Province were taken as the research objects. Through high-throughput sequencing technology, and with the help of PLS-DA and RDA, the impacts of rhizosphere soil microorganisms and environmental factors on the quality of *G. scabra* were explored in depth.

**Results:**

Alpha diversity shows that the diversity of bacterial communities varies significantly, while the regularity of fungi is weak; beta diversity shows that samples from different producing areas can be effectively grouped according to community structure. LDA effect shows that the differential species of bacteria and fungi vary among different producing areas. Indicator and random forest analysis show that *Sphingomonas* and *Subgroup_2* are the main indicator species of the bacterial communities in the high-content group, which can increase the evenness of microbial communities and maintain or enhance species diversity. The regularity of fungal communities is relatively weak. Functional metagenomic analysis shows that the functions of soil microorganisms in the six producing areas are similar but the relative abundances are different. The main functions of bacteria are closely related to microbial metabolism in diverse environments, biosynthesis of secondary metabolites, metabolic pathways, etc.; fungi are mainly lichen parasite, plant saprotroph, and ericoid mycorrhizal. PLS-DA and RDA analysis show that properly adjusting the key environmental factors of Ca, pH, and rapidly available potassium, which have a great influence on *G. scabra*, can affect the abundances of microorganisms such as *Subgroup_2*, *Burkholderia-Caballeronia-Paraburkholderia*, *Metarhizium*, *Bryobacter*, *Fusarium*, *Rhodanobacter*, *Cladophialophora*, *Sphingomonas* and *Trichoderma*, and then regulate the content of gentiopicroside.

**Discussion:**

This study provides practical microbial approaches and strategies for improving gentiopicroside content in the roots and rhizomes of *G. scabra*, and lays a solid scientific foundation for ensuring the quality and safety of genuine medicinal materials and the stable and sustainable development of the *G. scabra* planting industry.

## 1 Introduction

*Gentiana scabra* Bunge, a perennial herbaceous plant of the genus *Gentiana* (family Gentianaceae), is used medicinally with its roots and rhizomes. Traditionally, it is employed to clear heat, dry dampness, and purge liver-gallbladder fire (Lv et al., 2018). Modern pharmacological studies have demonstrated its hepatoprotective, analgesic, gastroprotective, antiviral, antitumor, and anti-inflammatory properties (Jia et al., 2022). It also exhibits therapeutic potential in treating cardiovascular and cerebrovascular diseases (Zhang L. et al., 2023), accelerating wound healing (Almukainzi et al., 2022), and relaxing smooth muscles (Waltenberger et al., 2015). However, overexploitation driven by increasing commercial demand has severely depleted wild *G. scabra* populations (Liu et al., 2021). To conserve this endangered species, artificial cultivation (primarily monoculture) was initiated in China during the early 1990s (Ling et al., 2017). Notably, cultivated *G. scabra* from geo-authentic producing areas (e.g., Liaoning Province) exhibits superior quality, efficacy, and consistency compared to wild counterparts (Liu et al., 2019). Gentiopicroside, a key bioactive marker specified (Chinese Pharmacopoeia Commission, 2020), serves as the primary quality indicator for *G. scabra*. Its accumulation (quantified in root tissues) is jointly affected by abiotic factors (geographical location and ecological conditions) (Reyes et al., 2019; Wang et al., 2020; Guo et al., 2013) and biotic factors. Among biotic factors, the rhizosphere microbial community plays a particularly significant role in mediating gentiopicroside-dependent soil microbial variability under monoculture systems.

Soil microorganisms, comprising both plant-growth-promoting and pathogenic taxa, serve as vital indicators of soil fertility and ecosystem health. Functioning as a “biobarometer” of terrestrial ecosystems, they drive essential processes such as plant metabolism, nutrient cycling, and organic matter decomposition. Notably, rhizosphere microbial communities often termed the plant’s “second genome” (Berendsen et al., 2012). It exerts profound influences on plant development and secondary metabolite synthesis through dynamic interactions with host plants. The structural composition, diversity, and abundance of rhizosphere microbiota critically regulate ecosystem functions, including carbon sequestration, soil mineralization, and productivity (Laforest-Lapointe et al., 2017; Bastida et al., 2018). These microbial traits not only shape plant growth but also modulate the accumulation of pharmacologically active compounds in medicinal species, particularly in monoculture environments where root exudate-microbe feedback is intensified. Advances in high-throughput sequencing technologies have revolutionized soil microbiome research by enabling cost-effective, large-scale profiling of microbial communities (Guan et al., 2013). This approach provides unprecedented insights into the functional potential of soil microorganisms and their responses to environmental variables, thereby facilitating targeted studies on microbe-environment interactions (Dang et al., 2019).

Liaoning Province is one of the main geo-authentic producing area for cultivating *G. scabra*, and the total amount of *G. scabra* medicinal materials supplied each year accounts for more than 83% of the Chinese *G. scabra* market (Rui et al., 2021). While monoculture enhances yield, it often leads to soil degradation, microbial community imbalance, and reduced biosynthesis of bioactive compounds like gentiopicroside, ultimately compromising the quality of authentic medicinal materials. Studying the rhizosphere soil microorganisms and environmental factors that cause differences in the gentiopicroside content of *G. scabra* in Liaoning Province, people can better control the quality of *G. scabra* medicinal materials and provide a guarantee for the effectiveness of clinical medication. Therefore, in this study, the rhizosphere soil and plants of *G. scabra* from different producing areas in Liaoning Province was collected. Analyze the content of gentiopicroside in the roots and rhizomes in relation to rhizosphere soil microorganisms and environmental factors, elucidate how soil microbial variability and environmental factors under monoculture conditions influence gentiopicroside accumulation in *G. scabra*, thereby identifying strategies to mitigate soil degradation and improve medicinal quality. The findings provide a scientific basis for optimizing the artificial cultivation of *G. scabra*, improving gentiopicroside content, and ensuring the sustainable development of authentic medicinal materials.

## 2 Materials and methods

### 2.1 Materials

#### 2.1.1 Main reagents

The gentiopicroside reference standard (Batch No. Y30J9Q66926) was purchased from Shanghai Yuanye Bio-Technology Co., Ltd., with a purity of ≥98%. Phosphoric acid was of analytical grade; methanol was of chromatographic grade (Merck KGaA); Wahaha purified water was obtained from Hangzhou Wahaha Group Co., Ltd. Potassium dichromate, concentrated sulfuric acid, ferrous sulfate, silicon dioxide, phenanthroline indicator, sodium hydroxide, and sodium pyrophosphate were all of analytical grade. Bacterial forward primer 343F (5′-TACGGRAGGCAGCAG-3′), bacterial reverse primer 798R (5′-AGGGTATCTAATCCT-3′), fungal ITS1 F (5′-CTTGGTCATTTAGAGGAAGTAA-3′) and ITS2 R (5′-GCTGCGTTCTTCATCGATGC-3′), 2% agarose, 5 × FastPfu buffer, 2.5 mmol/L dNTPs, DNA template, forward primer (5 μmol/L), FastPfu polymerase, BSA, ddH_2_O.

#### 2.1.2 Main instruments

Model 1,260 high-performance liquid chromatograph equipped with a diode array detector (DAD) (Agilent Technologies, United States)-PT-35SL micro-electronic balance (Huazhi Electronic Technology Co., Ltd.)-PHS-3E pH meter (Shanghai Yidian Scientific Instrument Co., Ltd.)-KQ5200DB CNC ultrasonic cleaner (Kunshan Ultrasonic Instruments Co., Ltd.)-FW100 high-speed universal grinder (Tianjin Taisite Instrument Co., Ltd.)-DHG-9145A blast drying oven (Shanghai Yiheng Scientific Instrument Co., Ltd.)-WKY II2 micropipette (Shanghai Jia’an Analytical Instrument Factory).

### 2.2 Sample collection

Methods based on the authenticity of the production areas, six representative *G. scabra* planting areas were selected for the collection of rhizosphere soils in September 2022. The longitude, latitude, altitude, aspect, and slope of the sampling points were determined by GPS to collect information about the collection sites. The five-point sampling method was adopted, and five sampling points were determined in a plum-blossom shape for each treatment. In each sampling point, well-grown *G. scabra* samples were selected. Approximately 10 g of *G. scabra* rhizosphere soil at a horizontal depth of 0–20 cm was collected as one sampling point using the shaking-off method. After all five sampling points were sampled, the soil samples were thoroughly mixed. Then, the uniformly mixed soil samples were selected by the quartering method and preserved as one soil sample to be tested. Three biological replicates were carried out for each production area. The samples to be tested were placed in sterile sealed bags and stored in foam boxes filled with dry ice. After all the samples were collected, they were transported back to the laboratory and stored in a −80°C low-temperature freezer for subsequent analysis of soil microorganisms.

### 2.3 Determination of gentiopicroside and environmental factors

The content of gentiopicroside in the roots and rhizomes of *G. scabra* was determined using high-performance liquid chromatography (HPLC). The chromatographic conditions were as follows: Agilent C18 column (4.6 mm × 250 mm, 5 μm), column temperature of 35°C, flow rate of 1 mL/min, detection wavelength of 270 nm, and isocratic elution with methanol-water (25:75) as the mobile phase. The injection volume was 10 μL, and the sampling time was 30 min. Based on the content of gentiopicroside in the roots and rhizomes of *G. scabra* not less than 30 mg/g as the standard, the production areas were divided into high-content production areas and low-content production areas (Table 1). The 30 mg/g threshold aligns with pharmacopeia grade criteria, enabling clinically meaningful group comparisons while retaining location-specific resolution for agronomic interventions. While the HPLC experiment is in progress, measure the following soil indicators according to their respective standards: available phosphorus (UV–visible spectrophotometry, NY/T 1121.7-2014), rapidly available potassium (atomic absorption spectrophotometry, LY/T 1234-2015), copper (flame atomic absorption spectrophotometry, HJ 491-2019), zinc (flame atomic absorption spectrophotometry, HJ 803-2016), manganese (aqua regia extraction-inductively coupled plasma mass spectrometry, NY/T 296-1995), calcium (atomic absorption spectrophotometry, NY/T 85-1988), magnesium (atomic absorption spectrophotometry), iron (atomic absorption spectroscopy), alkaline hydrolyzable nitrogen (alkaline diffusion method), soil organic matter and humus (potassium dichromate volumetric method), soil water content (drying method), and soil pH (potentiometric method). Fulvic acid, humic acid, humin, and the combination of humic acid and fulvic acid were determined using the NY/T 1867-2010 standard for the determination of soil humus composition. Through the data query system of the China Meteorological Science Data Sharing Service Network,^1^ local meteorological bureaus, and local statistical yearbooks, 11 meteorological factors, including average air pressure and annual average temperature, were screened out. In total, there were 33 environmental factors, including latitude, longitude, altitude, aspect, and slope.

**Table 1:** Six collection locations information.

Sample	Locality	Gentiopicroside (mg/g)
HGs-1	Shihugou Township, Kuandian County, Dandong City, Liaoning Province	64.2781
HGs-2	Qingyuan Town, Qingyuan County, Fushun City, Liaoning Province	52.8075
HGs-3	Hongmiaozi Township, Xinbin County, Fushun City, Liaoning Province	51.8782
HGs-4	Ying’emen Town, Qingyuan County, Fushun City, Liaoning Province	38.0465
LGs-1	Wangqingmen Town, Xinbin County, Fushun City, Liaoning Province	17.1984
LGs-2	Liangquan Town, Xifeng County, Tieling City, Liaoning Province	18.7285

### 2.4 Rhizosphere soil microbial molecular sequencing

#### 2.4.1 Rhizosphere soil bacterial molecular sequencing

In this study, the V3–V4 region of the bacterial 16S rRNA gene was used as the target DNA sequence for PCR amplification. The V3–V4 region of 16S rDNA was amplified using the forward primer 343F (5′-TACGGRAGGCAGCAG-3′) and the reverse primer 798R (5′-AGGGTATCTAATCCT-3′). The PCR amplification reaction system (20 μL) consisted of 2 μL of DNA template, 4 μL of 5 × FastPfu buffer, 2 μL of 2.5 mmol/L dNTPs, 1 μL of forward primer (5 μmol/L), 1 μL of reverse primer (5 μmol/L), 0.4 μL of FastPfu polymerase, 0.2 μL of BSA, and 9.4 μL of ddH₂O. The PCR reaction conditions were as follows: pre-denaturation at 95°C for 3 min; denaturation at 95°C for 30 s, annealing at 55°C for 30 s, and extension at 72°C for 45 s, for 25 cycles; and final extension at 72°C for 20 min. After the amplification was completed, 2% agarose gel electrophoresis was used to examine the amplification effect of the PCR amplification products. The PCR products of the samples were subjected to high-throughput sequencing on the Illumina MiSeq platform.

#### 2.4.2 Rhizosphere soil fungal molecular sequencing

The total DNA genome of each sample was extracted by the CTAB method. The extracted DNA was detected by 1% agarose gel electrophoresis, with three replicates for each sample. Using the diluted DNA as a template, PCR amplification was carried out with a high-fidelity enzyme. The primer sequences for amplification were: ITS1 F (5′-CTTGGTCATTTAGAGGAAGTAA-3′) and ITS2 R (5′-GCTGCGTTCTTCATCGATGC-3′). The PCR amplification reaction system (20 μL) consisted of 3 μL of DNA template, 4 μL of 5 × FastPfu buffer, 2 μL of 2.5 mmol/L dNTPs, 0.8 μL of forward primer (5 μmol/L), 0.8 μL of reverse primer (5 μmol/L), 0.4 μL of FastPfu polymerase, 0.2 μL of BSA, and 8.8 μL of ddH_2_O. The PCR reaction conditions were as follows: pre-denaturation at 97°C for 1 min; denaturation at 95°C for 10 s, annealing at 50°C for 30 s, extension at 72°C for 30 s, for 35 cycles; and final extension at 72°C for 4 min. The PCR products were detected by 2% agarose gel electrophoresis and recovered and purified using the gel recovery kit provided by Thermo Scientific. The qualified PCR products were used for Illumina MiSeq sequencing.

### 2.5 Data processing and analysis

The cutadapt software was used to cut off the primer sequences from the raw data sequences. The DADA_2_ was used to conduct quality - control analyses such as quality filtering, noise reduction, splicing and chimera removal on the qualified paired - end raw data from the previous step with the default parameters of QIIME2 to obtain the representative sequences and the ASV abundance table. ASV tables were rarefied to the minimum sequencing depth (54,116 for bacteria; 59,230 for fungi) to normalize sample read counts. QIIME_2_ (2020.11) (Bolyen et al., 2019) was used to deduplicate the sequences after DADA_2_ noise-reduction and chimera-removal. This method no longer clustered based on similarity, but only deduplicated, which was equivalent to clustering with 100% similarity. Each deduplicated sequence generated after quality-control was called ASVs (amplicon sequence variants, that is, characteristic sequences). The results of the detected microbial community structure were analyzed and plotted using the OE Cloud tools. The R package was used for LEfSe analysis, indicator analysis and randomForest analysis, and difference-species maps, indicator-species maps and species-importance point maps were drawn. PICRUSt2 (2.3.0b0) was used to predict the microbial functions. Excel 2019 was used to process the experimental data. The SIMCA 14.1 software was used to analyze the environmental factors of *G. scabra* in 33 different regions. The Canoco 5 software was used to conduct RDA analysis on the key environmental factors, the content of gentiopicroside and the microbial abundance.

## 3 Results

### 3.1 Quality control of sequencing data and evaluation of sequencing depth

**Figure 1:**
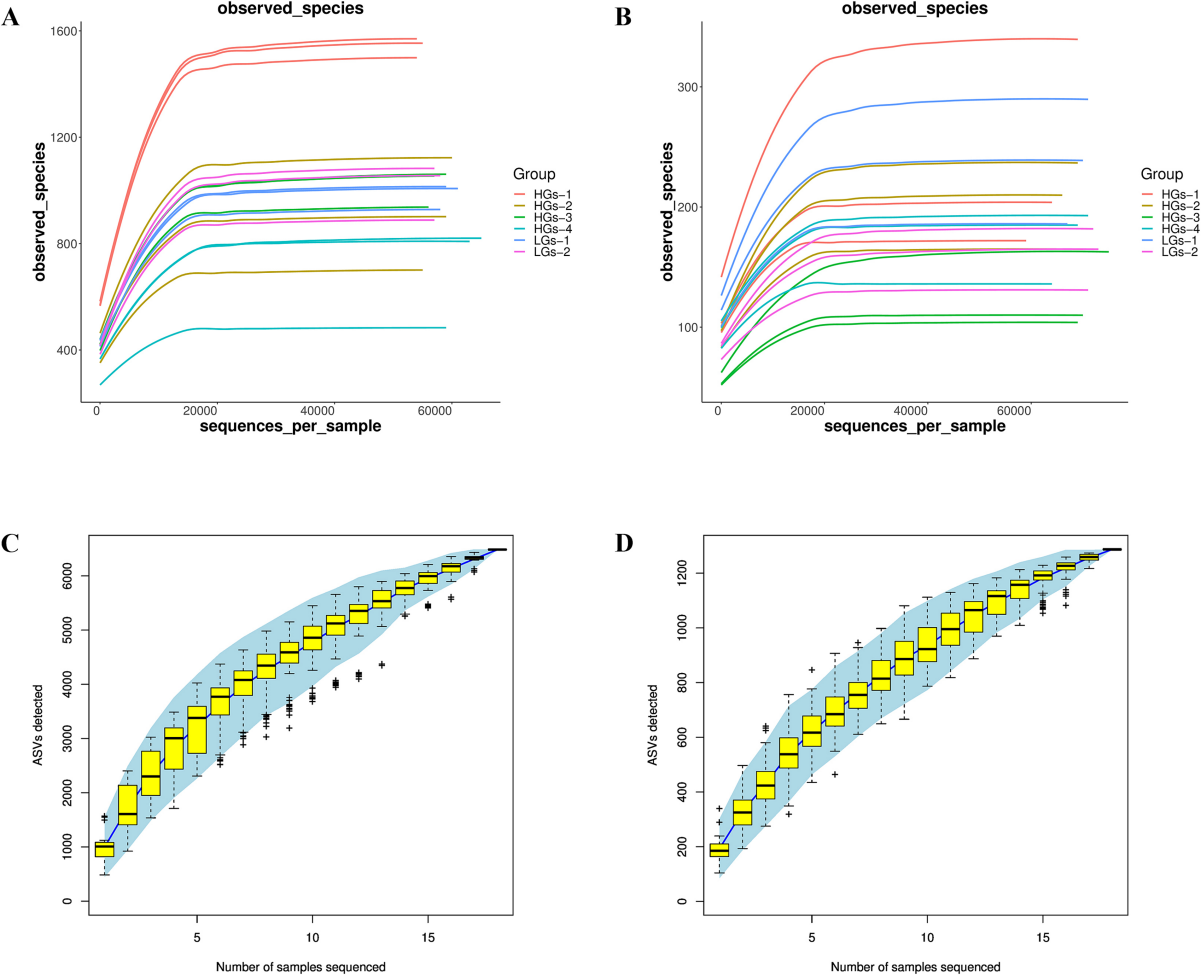
Rarefaction curves and Specaccum species accumulation curves of soil samples from six regions. The rarefaction curves of bacterial communities **(A)** and fungal communities **(B)**. The Specaccum species accumulation curves of bacterial communities **(C)** and fungal communities **(D)**.

Sequencing data were obtained from 18 samples. For bacteria, the amount of raw reads data after sequencing ranged from 78,116 to 81,878. After quality control, the amount of clean tags data ranged from 57,416 to 67,327. After removing chimeras from the clean tags, the amount of valid tags (i.e., the data finally used for analysis) ranged from 54,116 to 65,254, and the number of ASVs in each sample ranged from 484 to 1,572. For fungi, the amount of raw reads data after sequencing ranged from 78,060 to 81,606. After quality control, the amount of clean tags data ranged from 59,274 to 75,858. After removing chimeras from the clean tags, the amount of valid tags ranged from 59,230 to 75,505, and the number of ASVs in each sample ranged from 104 to 340. ASV abundance tables were rarefied to the minimum sequencing depth prior to diversity calculations and statistical modeling. The rarefaction curves (Figures 1A,B) indicated that as the sequencing depth increased, the rarefaction curves of bacteria and fungi tended to flatten, and the observable ASVs basically remained unchanged. The coverage rate was above 99.9%, suggesting that the experiment had obtained the vast majority of sample information, the amount of sequencing data was reasonable, and it could truly reflect the community composition of soil bacteria and fungi. The Specaccum species accumulation curves (Figures 1C,D) also proved this point.

### 3.2 Analysis of the composition of rhizosphere soil microbial communities

#### 3.2.1 Composition of microorganisms at the phylum level

**Figure 2:**
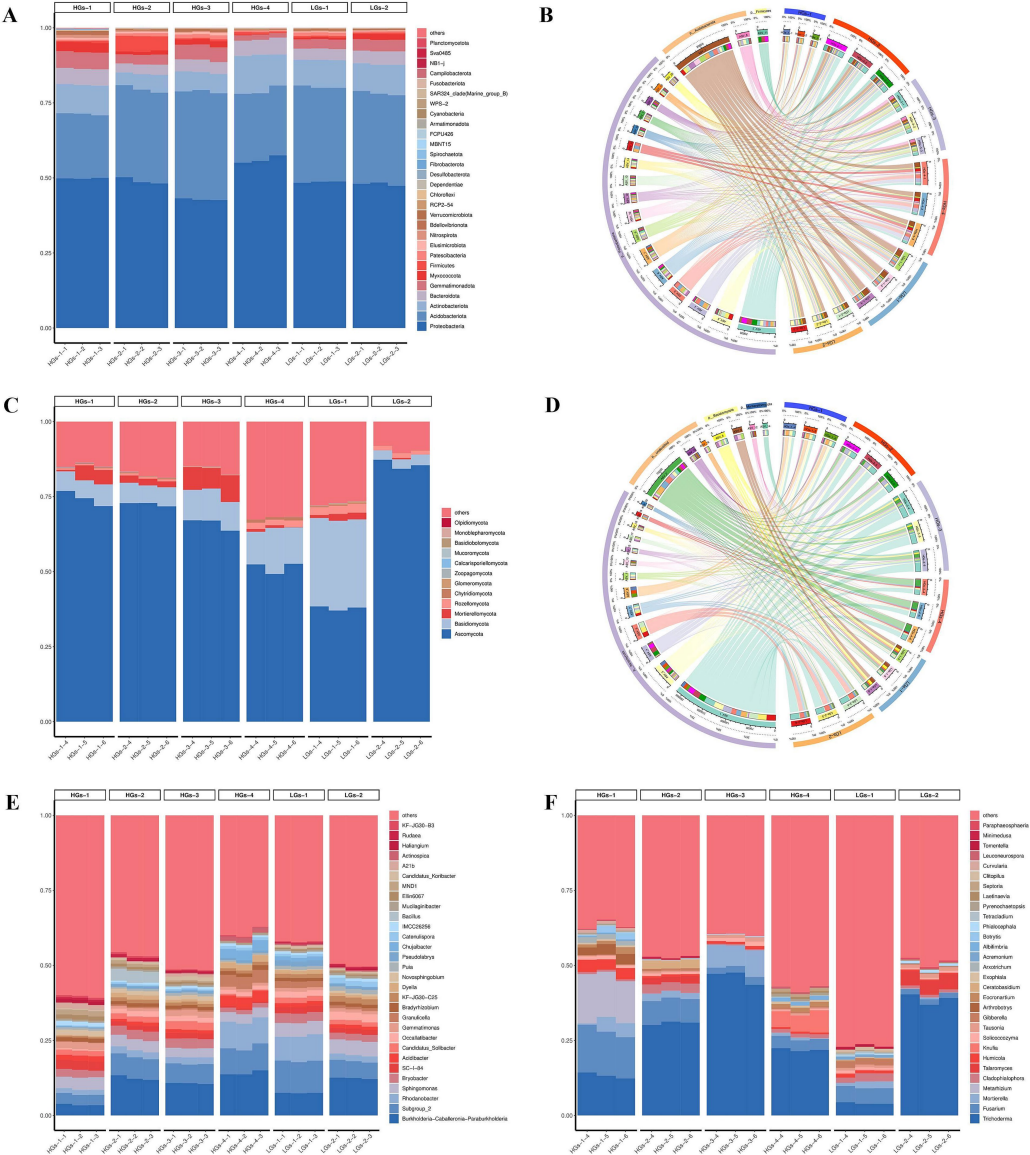
Relative abundances of the main bacterial communities at the phylum level in the soil **(A)** and the Circos diagram showing the relationship between samples and species **(B)**. Relative abundances of the main fungal communities at the phylum level in the soil **(C)** and the Circos diagram showing the relationship between samples and species **(D)**. Relative abundances of the main bacterial **(E)** and fungal **(F)** communities at the genus level (HGs-1, HGs-2, HGs-3, HGs-4, LGs-1, and LGs-2 represent codes for different locations, where the suffixes 1, 2, and 3 denote bacteria, and 4, 5, and 6 denote fungi).

When comparing the high-content group with the low-content group, it was found that the compositions of their top five microbial groups were similar, and only the relative abundances were different. The results of ASV annotation and relative abundance showed that in the bacterial community, the top five phyla in terms of relative abundance were Proteobacteria (42.85–56.09%), Acidobacteria (21.43–35.93%), Actinobacteriota (5.10–11.95%), Bacteroidota (3.19–5.38%), and Gemmatimonadota (1.69–5.55%) (Figure 2A). Proteobacteria and Acidobacteria had an absolute dominance in all bacterial communities (Figure 2B). In the fungal community, the top five phyla in terms of relative abundance were Ascomycota (37.84–85.68%), Basidiomycota (3.27–29.57%), Mortierellomycota (0.17–7.79%), Rozellomycota (0.10–2.79%), and Chytridiomycota (0.02–1.01%) (Figure 2C). Ascomycota had an absolute dominance in all fungal communities (Figure 2D).

#### 3.2.2 Composition of microorganisms at the genus level

At the genus level, the top five genera in terms of relative abundance in the bacterial community were *Burkholderia-Caballeronia-Paraburkholderia* (3.55–14.14%), *Subgroup_2* (3.49–10.62%), *Rhodanobacter* (1.66–8.80%), *Sphingomonas* (3.03–4.81%) and *Bryobacter* (1.46–3.16%) (Figure 2E). In the fungal community, the top five genera in terms of relative abundance were *Trichoderma* (4.05–46.11%), *Fusarium* (1.82–14.81%), *Mortierella* (0.17–7.79%), *Metarhizium* (0.01–15.16%) and *Cladophialophora* (0.39–3.00%) (Figure 2F).

#### 3.2.3 Analysis of the differences in the structures of bacterial and fungal communities in the rhizosphere soils of *Gentiana scabra* from different production areas

**Figure 3:**
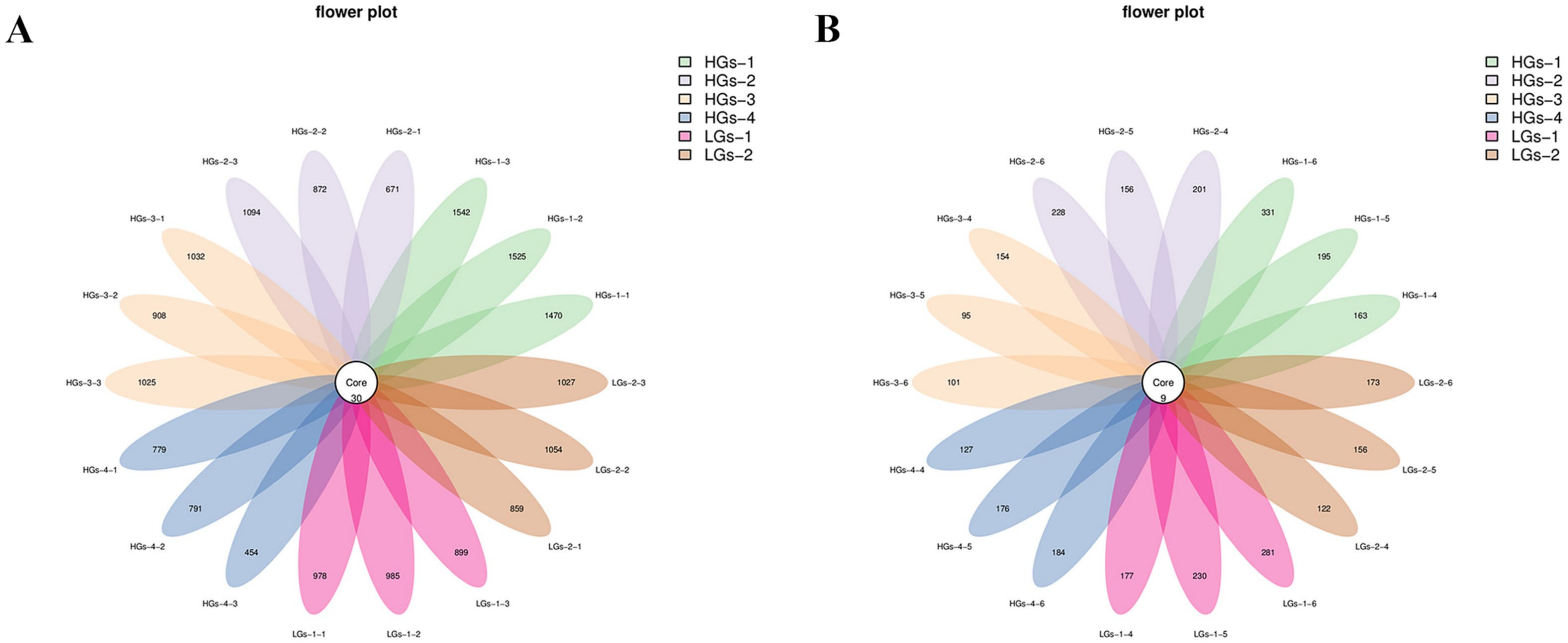
Petal diagrams of the distribution of ASVs of bacteria **(A)** and fungi **(B)** in the soil.

Petal diagrams were drawn based on the results of ASVs clustering analysis. In terms of the rhizosphere soil bacteria (Figure 3A), a total of 6,485 ASVs were obtained. There were 30 common ASVs among all the samples, and there were certain differences in the number of ASVs of rhizosphere soil microorganisms in different regions. The numbers of unique ASVs were 1,512 for HGs-1, 988 for HGs-3, 980 for LGs-2, 954 for LGs-1, 879 for HGs-2, and 675 for HGs-4. There were relatively large differences in the number of bacterial communities among samples from different production areas. On the whole, the number of ASV enrichments in the rhizosphere soils of the high-content group was greater than that of the low-content group. However, the number in HGs-4 was the lowest. In terms of the rhizosphere soil fungi (Figure 3B), a total of 1,287 ASVs were obtained. The number of common ASVs among the samples was 9. Among them, the number of unique ASVs was 230 for HGs-1, followed by 229 for LGs-1, 195 for HGs-2, 162 for HGs-4, 150 for LGs-2, and 117 for HGs-3. In the fungal community, there was no obvious relationship between the content groups and the number of ASV enrichments in the rhizosphere soil.

### 3.3 Analysis of the diversity of soil microbial communities

#### 3.3.1 Alpha diversity analysis

**Figure 4:**
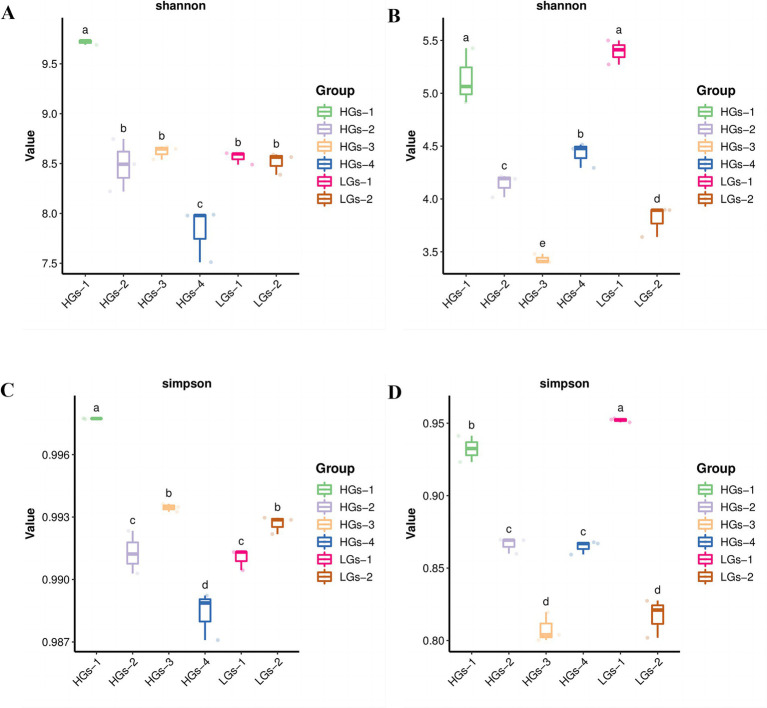
Shannon index diagrams of bacteria **(A)** and fungi **(B)** in the soil, and Simpson index diagrams of bacteria **(C)** and fungi **(D)** in the soil.

Alpha diversity analysis was conducted on the soils from six regions of *G. scabra*. According to the Shannon index (Figure 4A), the differences in the diversities of bacterial and fungal communities in the rhizosphere soils of *G. scabra* from different geographical locations were statistically significant (*p* < 0.05). The bacterial richness and evenness within the samples of HGs-1, HGs-2, and HGs-3 were relatively high, while the bacterial richness and evenness within the samples of LGs-1 and LGs-2 were relatively low. HGs-4 has the lowest alpha diversity. In terms of fungi (Figure 4B), there was no significant regularity. The results of the Simpson index in terms of diversity were highly consistent with those of the Shannon index (Figures 4C,D). Overall, there were many bacterial species in the HGs-1 group and they were evenly distributed, so the bacterial diversity was the highest; within the LGs-1 group, there were many fungal species and they were evenly distributed, so the fungal diversity was the highest.

#### 3.3.2 Beta diversity analysis

**Figure 5:**
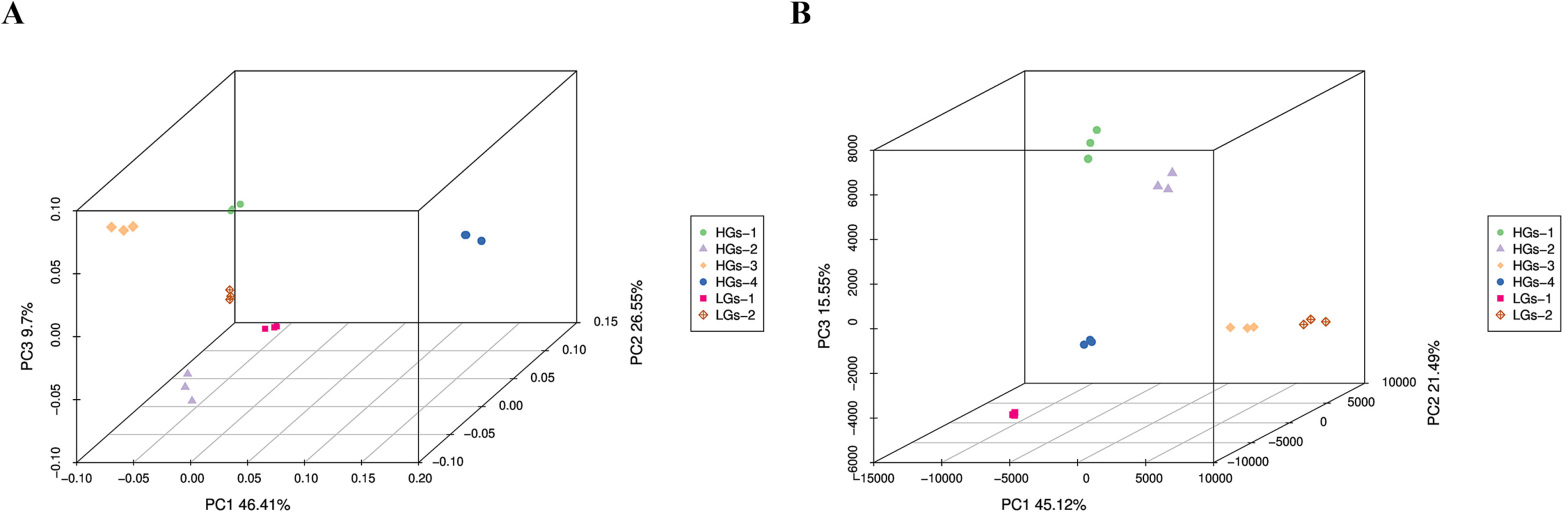
PCoA 3D plots of soil bacteria **(A)** and fungi **(B)** based on the weighted-UniFrac algorithm.

The results of the PCoA analysis of the bacterial community based on the weighted-UniFrac algorithm showed (Figure 5A) that the abscissa (PC1) and the ordinate (PC2) were the two main coordinates with the greatest explanatory power for the differences among samples, explaining 46.41 and 26.55% of the variance variation respectively, and their cumulative contribution rate was 72.96%. It can be seen from the figure that HGs-1, HGs-2, and HGs-3 were in one group, HGs-4 was in another group, and LGs-1 and LGs-2 were in one group. The samples within each group had community structures with similar compositions. The results of the PCoA analysis of the fungal community showed (Figure 5B) that the abscissa (PC1) and the ordinate (PC2) were the two main coordinates with the greatest explanatory power for the differences among samples, explaining 34.95 and 26.84% of the variance variation respectively, and their cumulative contribution rate was 61.79%. HGs-1 and HGs-2 were in one group, HGs-3 and HGs-4 were in another group, LGs-1 was in one group, and LGs-2 was in another group. The samples within each group had community structures with similar compositions.

### 3.4 Multivariate statistical analysis of microorganisms

**Figure 6:**
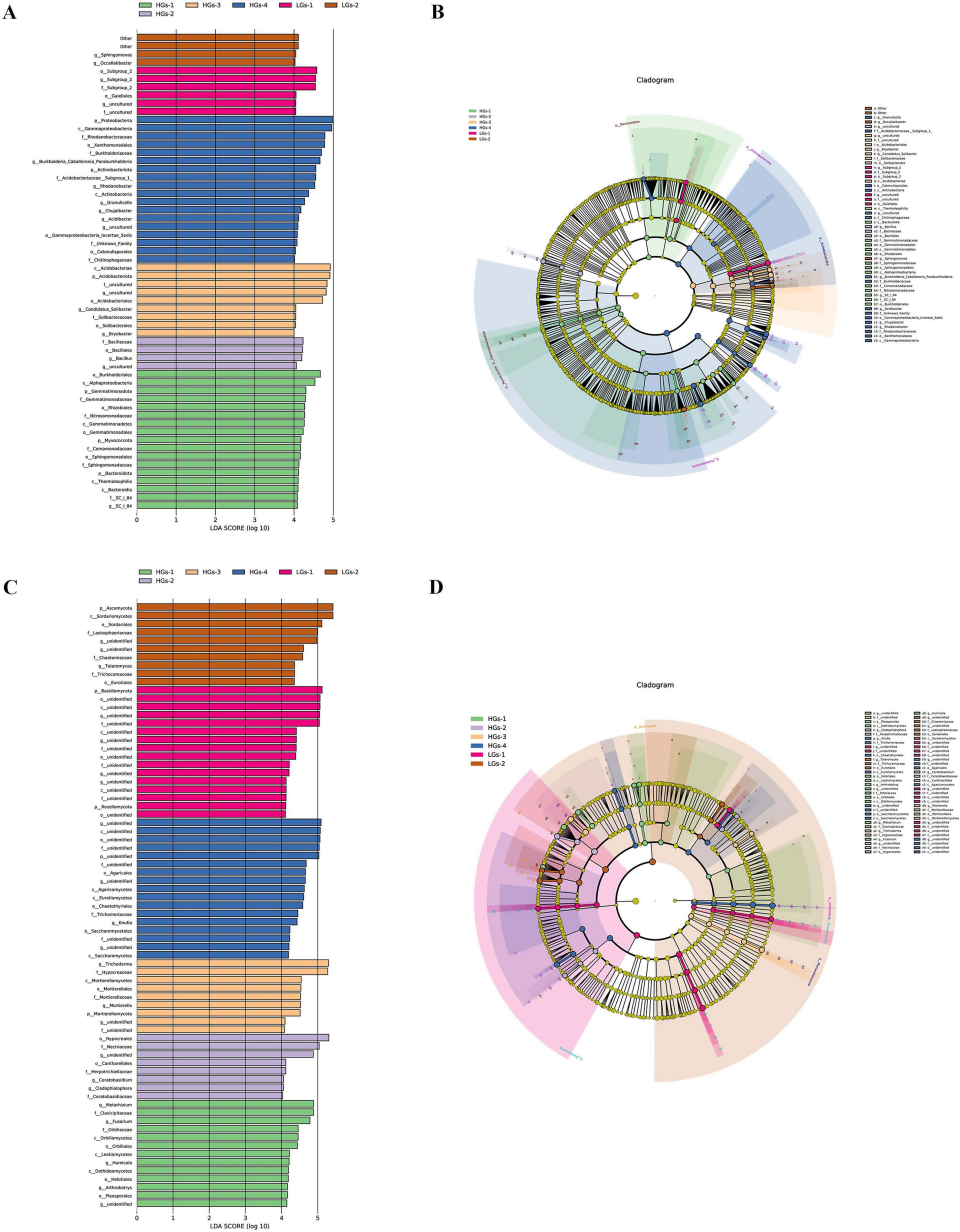
Score diagram of differential species of soil bacteria **(A)** and an example diagram of annotation branches of differential species **(B)**. Score diagram of differential species of soil fungi **(C)** and an example diagram of annotation branches of differential species of soil fungi **(D)**.

The analysis of the linear discriminant analysis (LDA) effect size for bacteria showed that HGs-1, HGs-2, and HGs-3 had more differential species with relatively higher bacterial relative abundances than LGs-1 and LGs-2, while HGs-4 the highest (Figure 6A). The proportions of Thermoleophilia, Bacteroidia, Gemmatimonadaceae, Gemmatimonadales, Gemmatimonadetes, Rhizobiales, Sphingomonadaceae, Sphingomonadales, Alphaproteobacteria, Comamonadaceae, Nitrosomonadaceae, *SC-I-84*, and Burkholderiales significantly increased in HGs-1. The proportions of *Bacillus*, Bacillaceae, and Bacillales significantly increased in HGs-2. The proportions of Acidobacteriales, *Bryobacter*, *Candidatus_Solibacter*, Solibacteraceae, Solibacterales and Acidobacteriae significantly increased in HGs-3. The proportions of *Granulicella*, Acidobacteriaceae_Subgroup_1, Catenulisporales, Actinobacteria, Chitinophagaceae, *Burkholderia_Caballeronia_Paraburkholderia*, Burkholderiaceae, *Acidibacter*, Gammaproteobacteria_Incertae_Sedis, *Chujaibacter*, *Rhodanobacter*, Rhodanobacteraceae, Xanthomonadales and Gammaproteobacteria significantly increased in HGs-4. The proportion of Gaiellales significantly increased in LGs-1. The proportions of *Occallatibacter* and *Sphingomonas* significantly increased in LGs-2 (Figure 6B).

The analysis of the linear discriminant analysis (LDA) effect size for fungi showed that there was no correlation between the differential species with relatively higher fungal relative abundances in the high-content group and those in the low-content group (Figure 6C). In the fungal community, the proportions of Pleosporales, Dothideomycetes, Helotiales, Leotiomycetes, _*Arthrobotrys*, Orbiliaceae, Orbiliales, Orbiliomycetes, *Metarhizium*, Clavicipitaceae, *Fusarium* and *Humicola* significantly increased in HGs-1. The proportions of *Cladophialophora*, Herpotrichiellaceae, Nectriaceae, Hypocreales, *Ceratobasidium*, Ceratobasidiaceae, and Cantharellales significantly increased in HGs-2. The proportions of *Trichoderma*, Hypocreaceae, *Mortierella*, Mortierellaceae, Mortierellales, and Mortierellomycetes significantly increased in HGs-3. The proportions of *Knufia*, Trichomeriaceae, Chaetothyriales, Eurotiomycetes, Saccharomycetales, Saccharomycetes, Agaricales, and Agaricomycetes significantly increased in HGs-4. There were no clear microorganisms whose proportions significantly increased in LGs-1. The proportions of *Talaromyces*, Trichocomaceae, Eurotiales, (unidentified-Rozellomycota), Chaetomiaceae, Lasiosphaeriaceae, Sordariales, and Sordariomycetes significantly increased in LGs-2 (Figure 6D).

### 3.5 Analysis of the differences in rhizosphere soil microbial species and indicator species

**Figure 7:**
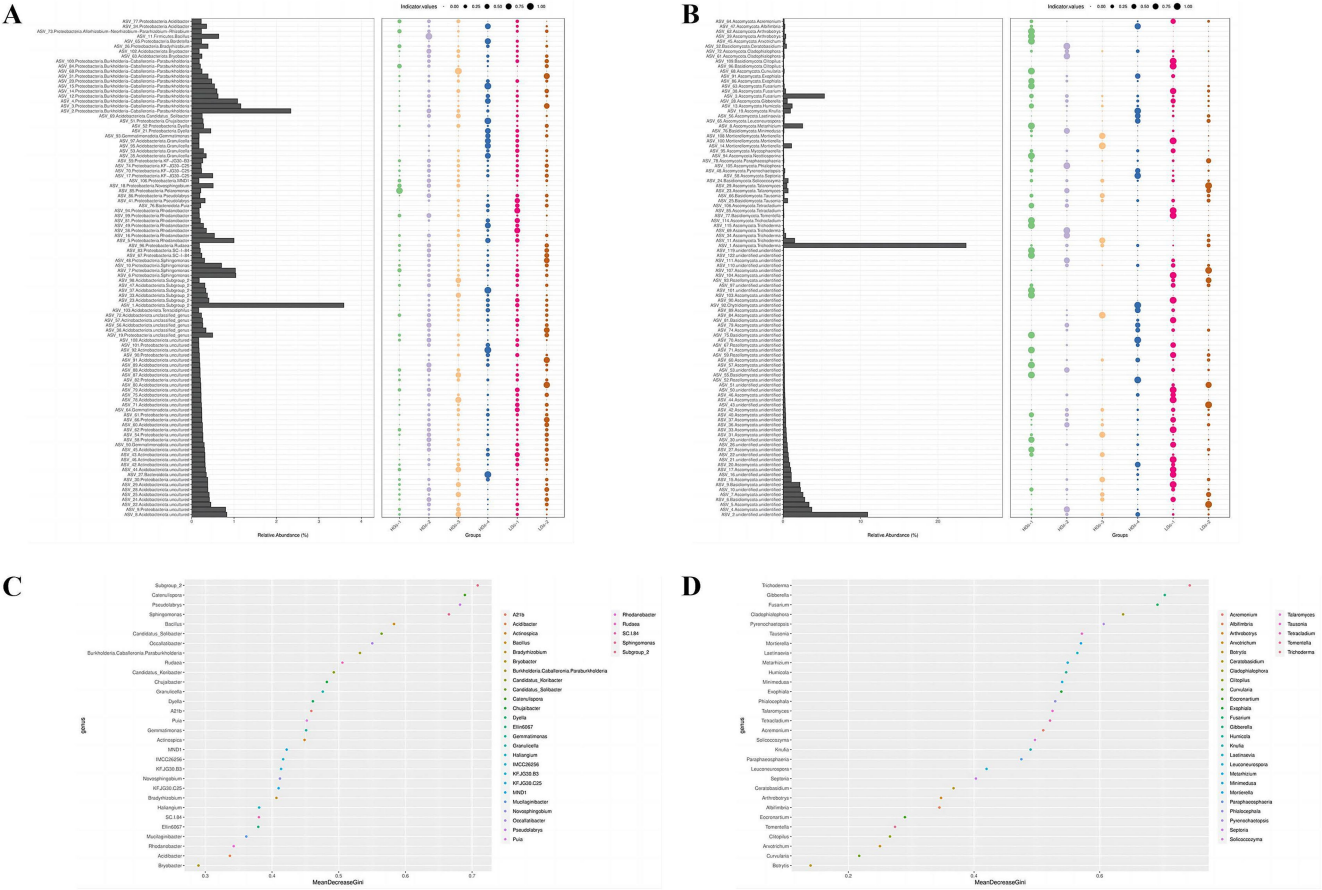
Indicator species diagrams of soil bacteria **(A)** and soil fungi **(C)**. Importance species point diagrams of soil bacteria **(B)** and soil fungi **(D)**.

Perform indicator analysis on the ASVs with the top 100 relative abundances and a statistical significance p less than 0.05. ASVs with an indicator value greater than 0.3 are regarded as major indicator species, and those with an indicator value greater than 0.7 are considered significant indicator species. Then, conduct visual analysis on the obtained results. The indicator analysis showed that these differences were quite significant at the genus level. In the bacterial community (Figure 7A), there were eight main indicator bacterial genera in HGs-1, and *Polaromonas* was a significant indicator species. There were 20 main indicator bacterial genera in HGs-2, and *Bacillus* was a significant indicator bacterial genus. There were 11 main indicator bacterial genera in HGs-3, and *Burkholderia-Caballeronia-Paraburkholderia* was a significant indicator bacterial genus. There were 21 main indicator bacterial genera in HGs-4, and *Acidibacter*, *Burkholderia-Caballeronia-Paraburkholderia*, *Subgroup*_*2* and *Chujaibacter* were significant indicator bacterial genera. There were 12 main indicator species in LGs-1, and *Rhodanobacter* was a significant indicator bacterial genus. There were 11 main indicator bacterial genera in LGs-2, and *Burkholderia-Caballeronia-Paraburkholderia* and *Sphingomonas* were significant indicator bacterial genera. In HGs-1, HGs-2, and HGs-3, *Acidibacter*, *MND1*, *Novosphingobium*, *Rhodanobacter*, *Sphingomonas* and *Subgroup*_*2* were the common main indicator bacterial genera. In LGs-1 and LGs-2, *Bryobacter*, *Burkholderia-Caballeronia-Paraburkholderia*, *Granulicella* and *Puia* were the common main indicator bacterial genera, and the main indicator bacterial genera of HGs-4 also existed in other groups. In the fungal community, although there were many indicator species, no common main indicator species could be found in the high-content group, and the same was true for the low-content group (Figure 7B).

The random forest analysis showed that in the bacterial community, based on the average reduction value of the Gini coefficient, *Subgroup_2*, *Catenulispora*, *Pseudolabrys* and *Sphingomonas* had good classification performance, indicating that the uniformity of these microbial communities increased and the species diversity was maintained or improved in the microbial community (Figure 7C). *Trichoderma*, *Gibberella*, *Fusarium*, *Cladophialophora* and *Pyrenochaetopsis* had good classification performance in the fungal community (Figure 7D).

### 3.6 Functional metagenomic analysis

**Figure 8:**
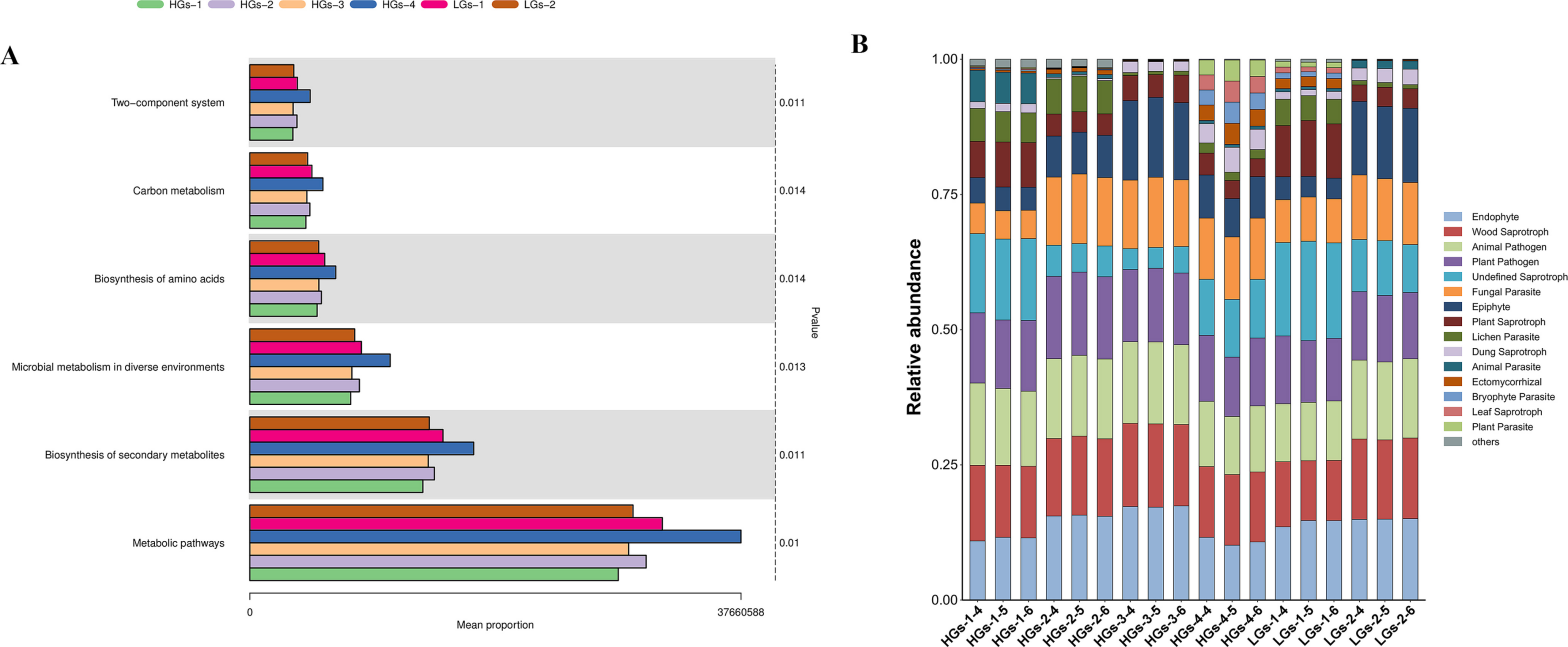
Bar chart of KEGG difference results for soil bacteria **(A)**. Histogram of FUNGuild for soil fungi **(B)**.

Based on the results of 16S rRNA amplicon sequencing, the microbiota of *G. scabra* was analyzed and the functional profiles of the bacterial core microbiota were predicted. The results of the functional metagenomic analysis showed that the microbial functions of soil samples from the six production areas were relatively similar, but there were certain differences in relative abundances. Hundreds of pathways commonly used for enrichment were utilized to predict the rhizosphere soil microorganisms of *G. scabra*, and a total of 372 pathways were detected. The main functions were concentrated on the two-component system, carbon metabolism, biosynthesis of amino acids, microbial metabolism in diverse environments, biosynthesis of secondary metabolites, and metabolic pathways. On the whole, the average abundance of pathways in the high-content groups is higher than that in the low-content groups, with HGs-4 being remarkably highest (Figure 8A). These functions are inextricably linked to the environment and secondary metabolites.

FUNGuild analysis was conducted on the fungal microbiota in the rhizosphere soil of *G. scabra* based on the sequencing results of ITS gene amplicon. The results showed that the fungal microorganisms in the rhizosphere soil of *G. scabra* were mainly concentrated in the lichen parasite of pathotroph (pathotroph), plant saprotroph of saprotroph (saprotroph, which grows in environments with many decaying plants), and ericoid mycorrhizal of symbiotroph (symbiotroph) (Figure 8B). Plant saprotroph is the main trophic mode in the fermentation process.

### 3.7 RDA analysis of microbial abundance, gentiopicroside and environmental factors

**Figure 9:**
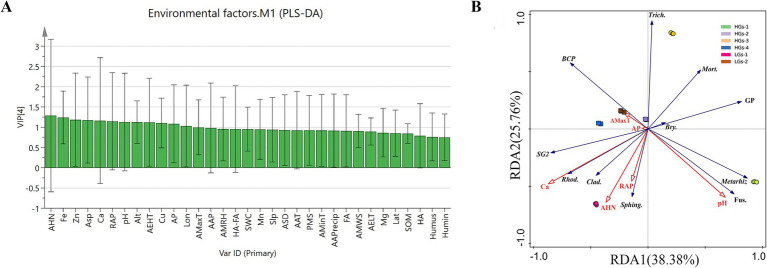
Distribution diagram of VIP values analyzed by the PLS-DA model **(A)** and the RDA analysis diagram **(B)**. Alt, altitude; Lon, longitude; Lat, latitude; Asp, aspect; Slp, slope; AAP, average air pressure; AMRH, annual mean relative humidity; AAT, annual average temperature; AMaxT, annual mean maximum temperature; AMinT, annual mean minimum temperature; AEHT, annual extreme high temperature; AELT, annual extreme low temperature; ASD, annual sunshine duration; AAPrecip, average annual precipitation; PMS, percentage of monthly sunshine; AMWS, annual mean wind speed; RAP, rapidly available potassium; AHN, alkaline hydrolyzed nitrogen content; AP, available phosphorus; SWC, soil water content; SOM, Soil organic matter; Humin, humin; Humus, humus; FA, fulvic acid; HA, humic acid; HA-FA, humic acid and fulvic acid; BCP, Burkholderia-Caballeronia-Paraburkholderia; SG2, Subgroup_2; Rhod., Rhodanobacter; Sphing., Sphingomonas; Bry., Bryobacter; Trich., Trichoderma; Fus., Fusarium; Mort., Mortierella; Metarhiz, Metarhizium; Clad., Cladophialophora; GP, gentiopicroside.

The information on environmental factors of the six production areas (Table 2) is as follows. In order to evaluate the impact of different environmental factors on *G. scabra*, 33 environmental factors were subjected to PLS-DA using SIMCA 14.1 (Figure 9A) (Miao et al., 2023). The VIP value represents the contribution of variables to the indicators. Environmental factors with VIP values >1 and greater contributions to the indicators were selected. The results showed that a total of 13 environmental factors, namely alkaline hydrolyzed nitrogen content, Fe, Zn, aspect, Ca, rapidly available potassium, altitude, Cu, pH, annual extreme high temperature, annual mean maximum temperature, longitude, and available phosphorus, had a greater impact on *G. scabra*. Based on the environmental factors obtained from PLS-DA, RDA analysis was carried out on gentiopicroside and the abundances of dominant microorganisms. Environmental factors with small pseudo-*F* values and without significance were gradually removed. The results are shown in Figure 9B. The first two axes explained 38.38 and 25.76% of the variance, respectively. The environmental factors that had the greatest impact on the microbial abundance of *G. scabra* and the accumulation of gentiopicroside were Ca, pH and rapidly available potassium (*p* < 0.01). The relative abundance of Subgroup_2 was significantly positively correlated with Ca, while the relative abundance of *Mortierella* was significantly negatively correlated with Ca. The relative abundance of *Burkholderia-Caballeronia-Paraburkholderia* was significantly negatively correlated with pH, while the relative abundances of *Metarhizium*, *Bryobacter* and *Fusarium* were significantly positively correlated with pH. The relative abundances of *Rhodanobacter* and *Cladophialophora* were significantly positively correlated with Ca and rapidly available potassium, the relative abundance of *Sphingomonas* was significantly positively correlated with rapidly available potassium, and the relative abundance of *Trichoderma* was significantly negatively correlated with rapidly available potassium. The relative abundances of *Mortierella*, *Trichoderma*, *Metarhizium*, *Bryobacter* and *Fusarium* were significantly positively correlated with the content of gentiopicroside, while the relative abundances of *Subgroup_2*, *Rhodanobacter* and *Cladophialophora* were significantly negatively correlated with the content of gentiopicroside. The content of gentiopicroside was significantly positively correlated with pH and significantly negatively correlated with Ca and rapidly available potassium.

**Table 2:** Environmental factor data of *G. scabra* from six production areas.

Sample	HGs-1	HGs-2	HGs-3	HGs-4	LGs-1	LGs-2
Average air pressure (hPa)	98.55	98.83	97.79	98.83	97.79	99.23
Annual average temperature (°C)	7.5	6.2	5.6	6.2	5.6	5.4
Annual mean maximum temperature (°C)	13.5	13.5	13.1	13.5	13.1	12.8
Annual mean minimum temperature (°C)	2.2	0.3	−0.6	0.3	−0.6	−1.0
Annual extreme high temperature (°C)	36.5	37.2	37.0	37.2	37.0	36.7
Annual extreme low temperature (°C)	−34.0	−36.3	−38.3	−36.3	−38.3	−43.4
Annual mean relative humidity (%)	71.00	70.00	72.00	70.00	72.00	69.00
Average annual precipitation (mm)	1077.80	780.80	776.60	780.80	776.60	692.20
Annual mean wind speed (m/s)	1.30	1.60	1.20	1.60	1.20	2.00
Annual sunshine duration (h)	2262.70	2254.40	2254.40	2254.40	2254.40	2618.30
Percentage of monthly sunshine (%)	51.00	51.00	51.00	51.00	51.00	59.00
Available phosphorus (g/kg)	2.00	118.40	52.00	95.90	39.80	61.20
Rapidly available potassium (g/kg)	150.00	158.00	133.00	17.00	306.00	206.00
Cu (mg/kg)	12.00	11.00	23.00	17.00	20.00	14.00
Zn (mg·kg)	63.00	48.00	81.00	55.00	74.00	65.00
Mn (mg/kg)	492.00	390.00	352.00	453.00	364.00	575.00
Ca (g/kg)	0.40	1.60	0.40	3.10	4.00	2.40
Mg (g/kg)	0.30	0.20	3.90	0.10	16.70	24.30
Fe (g/kg)	39.40	27.40	44.70	30.80	20.80	21.70
Soil water content (%)	7.60	3.30	5.30	3.60	5.20	3.00
Alkaline hydrolyzed nitrogen content (mg/kg)	84.00	55.30	79.10	79.10	200.20	65.10
Soil organic matter (%)	6.70	5.20	5.80	5.70	2.40	1.00
Humus (%)	2.00	0.90	1.40	1.30	1.10	0.40
Humic acid (g/kg)	0.90	0.90	0.70	0.70	0.50	0.50
Fulvic acid (g/kg)	0.80	0.70	1.00	0.60	1.30	1.80
Humic acid and fulvic acid (g/kg)	1.70	1.60	1.70	1.30	1.80	2.30
Humin (g/kg)	18.50	7.40	12.20	11.70	8.80	1.90
pH	5.60	4.80	4.50	4.70	4.80	5.00
Latitude (°)	40.73	42.10	41.54	42.18	41.74	42.73
Longitude (°)	124.82	124.92	125.15	125.09	125.04	124.72
Altitude (m)	341.04	238.96	472.30	305.61	314.53	191.57
Aspect (1)	5.72	6.34	6.19	5.49	7.40	6.00
Slope (°)	6.98	2.63	3.89	1.51	1.64	1.96

## 4 Discussion

Previous studies have shown that changes in the diversity or activities of the rhizosphere soil microbial communities of plants can significantly affect plant growth and environmental adaptation (Vandenkoornhuyse et al., 2015). Microbial communities usually vary depending on plant species or varieties, and the structures of microbial communities also depend on many environmental factors, such as climate, water, and biological interactions (Fortin Faubert et al., 2022).

### 4.1 Ecological significance of the analysis on the composition and diversity of the rhizosphere soil microbial community of *Gentiana scabra*

In the compositional analysis at the phylum level of rhizosphere soil microorganisms, the similarity in the main microbial groups between the high-content and low-content groups indicates that these microorganisms have a relatively stable status in the rhizosphere ecosystem of *G. scabra*. The dominant positions of Proteobacteria and Acidobacteria in the bacterial community have been reported in many studies on soil microorganisms (Fierer and Jackson, 2006; Lauber et al., 2009). Proteobacteria is widely involved in the nitrogen cycling process in the soil (Zehr et al., 2003). Acidobacteria has a special adaptability in acidic soil environments, and its relative abundance may be related to the acidic conditions of the soil (Kielak et al., 2016). In the fungal community, the absolute dominance of Ascomycota reflects its key functions in the soil ecosystem. Ascomycota can decompose complex organic substances, release nutrients, promote the improvement of soil fertility and nutrient cycling, and thus create favorable soil conditions for the growth of *G. scabra* (Challacombe et al., 2019). Moreover, differences in soil nutrient contents provide different growth resources for bacteria. The production areas of the high-content group may have richer nutrient conditions, which are beneficial to the growth and reproduction of bacteria and further increase the number of ASV enrichments (van der Heijden et al., 2008). In addition, climatic conditions such as temperature, precipitation, and wind speed also play a role in the formation of the bacterial community structure. Temperature affects the metabolic rate of bacteria, while precipitation and wind speed affect the water content and air permeability of the soil. These factors jointly shape the bacterial community structures in different production areas (Bei et al., 2023).

At the genus level, the biocontrol function of the genus *Burkholderia* in the bacterial community is of great significance for protecting *G. scabra* from pathogen attacks. Previous studies have shown that some strains of the genus Burkholderia can produce antibacterial substances or induce systemic resistance in plants to inhibit the growth of pathogens (Compant et al., 2005). The genus *Sphingomonas* plays a positive role in maintaining the quality of the rhizosphere soil environment and can reduce the negative impact of pollutants on the growth of *G. scabra* (Deng et al., 2022). In the fungal community, the genus *Trichoderma* can inhibit pathogens through multiple mechanisms to ensure the healthy growth of *G. scabra* (Harman et al., 2004). The genus *Fusarium* may form competitive or symbiotic relationships with other microorganisms and jointly affect the growth environment of *G. scabra* (Jin et al., 2024). There is no obvious relationship between the content groups in the fungal community and the number of ASV enrichments in the rhizosphere soil, which highlights the uniqueness of the response of the fungal community to environmental factors. There are differences in the types and quantities of substances such as sugars, amino acids, and organic acids secreted by the roots of *G. scabra* plants from different production areas. These secretions, as carbon sources and signal molecules for fungi, selectively promote or inhibit the growth of certain fungi, thus affecting the number and structure of ASVs in the fungal community (Bais et al., 2006). It should be noted that some strains of Burkholderia may be pathogenic to plants or humans. In this study, strain-specific functional verification was not carried out, and its actual ecological functions need to be further analyzed in combination with metagenomic data. The ecological role of *Fusarium* is highly dependent on the strain type. In the future, its functional differentiation can be clarified through isolation and culture.

The results of alpha diversity analysis showed that there were significant differences in the diversities of bacterial and fungal communities in the rhizosphere soils of *G. scabra* from different production areas. In terms of bacteria, the relatively high bacterial richness and evenness within the samples of HGs-1, HGs-2, and HGs-3 might be attributed to the more favorable environmental conditions in these production areas. These conditions provided a good environment for the growth and reproduction of bacteria, resulting in rich bacterial species and an even distribution, which was consistent with the previous research results on the relationship between microbial diversity and environmental adaptability (Bell et al., 2005). The regularity of the diversity of the fungal community was relatively weak, which might be due to the fact that the response of fungi to environmental factors was more complex and was comprehensively affected by multiple factors including soil conditions (Tedersoo et al., 2014). The results of beta diversity analysis indicated that samples from different production areas could be divided into different groups according to the structures of bacterial and fungal communities, and the samples within each group had similar community structures. This result further confirmed the role of environmental factors in shaping the structures of microbial communities and reflected the co-evolutionary relationship between the structures of microbial communities and the environment (Jansson and Hofmockel, 2020). When the rhizosphere soil changes from the “bacterial type” with high fertility to the “fungal type” with low fertility, the yield and quality will be reduced. Therefore, we should, based on the state of soil microorganisms, take targeted measures such as using microbial fertilizers to regulate the soil microbial environment so as to achieve the effects of increasing production and improving quality (Teng et al., 2024).

### 4.2 Ecological connotations of multivariate statistical analysis of microorganisms

The results of the linear discriminant analysis (LDA) effect size analysis for bacteria showed that the differential species with relatively higher bacterial abundances differed among different production areas, which reflected the selective effect of environmental conditions in different production areas on the composition of the bacterial community. These differential species have different functions in the soil ecological processes, such as participating in specific material cycling processes or forming special interaction relationships with *G. scabra* plants. Judging from the results, on the whole, the number of differential species in the high-content groups is greater than that in the low-content groups (except for HGs-4). This indicates that the more differential bacterial species there are, the more positive the impact on gentiopicroside in *G. scabra*. The analysis of the LDA effect size for fungi indicated that there was no obvious correlation between the differential species with relatively higher fungal abundances in the high-content group and those in the low-content group, which once again emphasized the complexity of the response of the fungal community to the environment. The growth and distribution of fungi are comprehensively affected by multiple factors, and the changes in these factors among different production areas have led to complex changes in the structure of the fungal community, which is consistent with the previous discussions on the influencing factors of the structure of the fungal community (Zeng et al., 2023). However, the differential species of fungi have no significant impact on *G. scabra* in different groups.

### 4.3 Ecological significance of the analysis on the differences in rhizosphere soil microbial species and indicator species

The indicator analysis showed significant differences at the genus level. In the bacterial community, different production areas had their respective main indicator bacterial genera, which provided important indications for in-depth understanding of the characteristics of bacterial communities in different production areas and their relationships with the content of gentiopicroside. These indicator bacterial genera might have formed special symbiotic or interaction relationships with *G. scabra* under specific production area environments, having a potential impact on the growth of *G. scabra* and the synthesis of secondary metabolites. In the fungal community, no common main indicator species were found in either the high-content or low-content groups, which further reflected the complexity of the fungal community structure and its greater stability to the environment (Yu et al., 2021). In the results of the random forest analysis, bacteria such as *Subgroup_2* and *Catenulispora* played important roles in maintaining the uniformity and diversity of the community. They might have participated in the processes of material cycling and energy flow in the soil and had a positive significance for the stability of the growth environment of *G. scabra* (Zhang et al., 2024; Zuo et al., 2022). In the fungal community, fungi such as *Trichoderma* and *Gibberella* had important positions in the rhizosphere ecosystem and might affect the rhizosphere soil environment and the growth of *G. scabra* by inhibiting pathogens and decomposing organic substances (Xu et al., 2022). The limitations of current research techniques make it difficult to accurately identify *Subgroup_2*’s taxonomic position. However, considering its ecological significance, we believe that retaining *Subgroup_2* can provide new perspectives for future research. By highlighting its potential importance, we hope to attract more attention from the scientific community and encourage further exploration of this unclassified bacterial subgroup.

### 4.4 Implications of functional metagenomic analysis for ecosystem functions

Plant secondary metabolites play a very important role in plant interactions and plant defense (Bais et al., 2002). The results of functional metagenomic analysis showed that the microbial functions of soil samples from six production areas were relatively similar, although there were certain differences in abundances. The main functions were concentrated on the two-component system, carbon metabolism, biosynthesis of amino acids, microbial metabolism in diverse environments, biosynthesis of secondary metabolites, and metabolic pathways. This indicates that although there are differences in the structures of microbial communities in different production areas, microorganisms have a certain degree of conservatism in basic life activities and ecological functions. These functions are closely related to the environment and secondary metabolites. For example, carbon metabolism is a key link in the energy flow of the soil ecosystem, affecting soil fertility and the plant growth environment (Griffiths and Philippot, 2013). Biosynthesis of amino acids not only provides essential substances for the growth of microorganisms themselves but may also interact with the protein synthesis and secondary metabolite synthesis pathways in *G. scabra*, jointly affecting the growth and development of *G. scabra* (Matulich et al., 2015). The relative abundances of bacterial microbial functions are highly consistent with the findings of previous studies. Basically, it can be shown that the higher the relative abundance of bacterial microorganisms, the higher the content of gentiopicroside in plants. Fungi play an important role in maintaining the material cycling of the ecosystem, soil quality, the stability of the structure and functions of biological communities, influencing biodiversity, promoting plant growth, and improving the ability of plants to cope with environmental pressures.

### 4.5 RDA analysis of microbial abundance, gentiopicroside and environmental factors

PLS-DA analysis was used to identify 13 environmental factors that had a significant impact on *G. scabra*, such as alkaline hydrolyzed nitrogen, iron, zinc, etc. As an important available nitrogen source for plants, the content of alkaline hydrolyzed nitrogen regulates the synthesis of secondary metabolites by influencing the growth and development of *G. scabra* (He et al., 2024). Soil pH is the most critical factor affecting the composition of the bacterial community (Meng et al., 2024; Ming et al., 2023; Wang et al., 2023; Shu et al., 2023). It is extremely significantly negatively correlated with alkaline hydrolyzed nitrogen, and most bacteria have limited pH tolerance (Pan et al., 2023; Rousk et al., 2010; Fernández-Calviño et al., 2011). Fe and Zn participate in the formation of the active centers of plant enzymes and regulate the activities of key enzymes in secondary metabolism (Marschner, 1995). Aspect is associated with the percentage of sunshine, and they jointly affect the light conditions of the plant growth environment. Geographical factors such as altitude and longitude indirectly affect the content of gentiopicroside (Flexas et al., 2008). Altitude is a key factor affecting bacterial diversity (Zhang Y. et al., 2023). Our research group previously found that rapidly available potassium is one of the dominant ecological factors affecting the quality of *G. scabra*, and its appropriate application is beneficial to the accumulation of dry matter (Li et al., 2022; Wei et al., 2018). Ca (Shabtai et al., 2023) and Cu can change the structure of the soil microbial community and promote the accumulation of secondary metabolites. The annual extreme high temperature can promote microorganisms to adjust their metabolic pathways, and the average air pressure indirectly affects the accumulation of secondary metabolites. RDA analysis further screened out that Ca, pH and rapidly available potassium are the key environmental factors affecting the microbial abundance of *G. scabra* and the accumulation of gentiopicroside. They significantly influence the accumulation of gentiopicroside by regulating the abundances of key microorganisms such as *Subgroup_2* and *Burkholderia-Caballeronia-Paraburkholderia*. This result validates the hypothesis of the coordinated regulation of secondary metabolite synthesis by microorganisms and environmental factors, providing direct evidence for the targeted regulation of the soil micro - environment to improve the quality of medicinal materials. Moreover, this study found that the higher the predicted relative abundance of bacteria in the rhizosphere soil of *G. scabra*, the higher the content of gentiopicroside it contained. Therefore, increasing the relative abundance of bacteria in the rhizosphere soil of *G. scabra* is beneficial for *G. scabra* to accumulate secondary metabolites. RDA analysis shows that meteorological and geographical factors have the least impact on microorganisms and gentiopicroside. This may be because the information on meteorological and geographical factors at the six sampling sites is relatively similar, and the significant differences in soil physical and chemical properties overshadow the influence of meteorological and geographical factors. In fact, these factors may play a more important role. Therefore, future research could further investigate the independent effects of soil factors and non-soil factors to clarify the indirect effects of meteorological and geographical factors.

Judging from the analysis results of the full text, on the whole, the high-content groups are significantly different from the low-content groups (except for HGs-4). It likely stems from its unique environmental conditions: soil chemistry: HGs-4 had the highest Ca (3.1 g/kg) and lowest rapidly available potassium (17 g/kg) among the high-content groups calcium dominance can inhibit bacterial growth through cation competition. Management practices: as a transition zone between high/low-content areas, HGs-4’s intermediate pH (4.7) and altitude (305.6 m) may create suboptimal conditions for microbial niche partitioning.

## 5 Conclusion

In summary, this paper conducted an in-depth analysis of the composition, structure, diversity, and functions of the rhizosphere soil microbial communities of *G. scabra* from different production areas, and identified the environmental factors that have a significant impact on *G. scabra*. The results showed that the differences in the structures of soil microbial communities were the result of the combined effects of multiple environmental factors, and the environmental factors in different production areas had an important impact on the content of gentiopicroside. The responses of bacterial and fungal communities to environmental factors had different characteristics. The alpha diversity and beta diversity analyses revealed the differences in microbial communities from different production areas and the co-evolutionary relationship with the environment. The multivariate statistical analysis of microorganisms and the functional metagenomic analysis further explained the ecological connotations of microbial communities and the implications for ecosystem functions. This study found that the microbial abundances in the rhizosphere soil of *G. scabra* and environmental factors could promote the accumulation of gentiopicroside in *G. scabra*. The research results provided a scientific basis for ensuring the safe and effective cultivation of genuine regional medicinal materials, the development of specialized microbial fertilizers, and the regulation of the soil microbial environment to achieve the effects of increasing gentiopicroside content.

## Data Availability

The data presented in the study are deposited in the NCBI Sequence Read Archive (SRA), accession number PRJNA1229856.

## Footnotes

^1^ http://data.cma.cn/data
